# Whole-Body MRI vs. PET/CT for the Detection of Bone Metastases in Patients With Prostate Cancer: A Systematic Review and Meta-Analysis

**DOI:** 10.3389/fonc.2021.633833

**Published:** 2021-05-04

**Authors:** Yuefu Zhan, Guangming Zhang, Mingliang Li, Xiaobo Zhou

**Affiliations:** ^1^West China Biomedical Big Data Center, West China Hospital, Sichuan University, Chengdu, China; ^2^Department of Radiology, Hainan Women and Children's Medical Center, Hainan, China; ^3^School of Biomedical Informatics, The University of Texas Health Science Center at Houston, Houston, TX, United States

**Keywords:** prostate cancer, magnetic resonance imaging, positron emission tomography, computed tomography, bone metastasis, meta-analysis

## Abstract

**Purpose:** A recent meta-analysis in patients with non-small cell lung cancer showed no difference between whole-body magnetic resonance imaging (WBMRI) and positron emission tomography/computed tomography (PET/CT), but no such study is available for prostate cancer (PCa). This study aimed to compare WBMRI and PET/CT for bone metastasis detection in patients with PCa.

**Materials and Methods:** PubMed, Embase, and the Cochrane library were searched for papers published up to April 2020. The population was the patients with untreated prostate cancer diagnosed by WBMRI or PET/CT. The outcomes were the true positive and negative and false positive and negative rates for WBMRI and PET/CT. The summarized sensitivity, specificity, positive likelihood ratios (PLR), negative likelihood ratios (NLR), and diagnostic odds ratios (DOR) were calculated with their 95% confidence intervals (CIs).

**Results:** Four prospective and one retrospective study are included (657 patients). Significant differences are observed between WBMRI and PET/CT for sensitivity (WBMRI/PET/CT: 0.896; 95% CI: 0.813–0.987; *P* = 0.025) and NLR (WBMRI/PET/CT: 2.38; 95% CI: 1.13–5.01; *P* = 0.023), but not for specificity (WBMRI/PET/CT: 0.939; 95% CI: 0.855–1.031; *P* = 0.184) and PLR (WBMRI/PET/CT: 0.42; 95% CI: 0.08–2.22; *P* = 0.305). WBMRI has a similar a DOR compared with PET/CT (WBMRI/PET/CT: 0.13; 95% CI: 0.02–1.11; *P* = 0.062). The summary area under the receiver operating characteristic curves for WBMRI is 0.88 (standard error: 0.032) and 0.98 (standard error: 0.013) for PET/CT for diagnosing bone metastases in PCa.

**Conclusion:** PET/CT presents a higher sensitivity and NLR for the bone metastasis detection from PCa, whereas no differences are found for specificity and PLR, compared with WBMRI.

## Introduction

Prostate cancer (PCa) is the most common cancer in males and among the most lethal cancers in men worldwide (1, 2, 12). About 10% of patients with PCa have bone metastasis at presentation, with a rate as high as 80% for patients with advanced PCa (3, 4), and about 33% of the remaining patients will develop metastases during follow-up (5, 6). In addition, patients presenting small numbers of metastases have a better prognosis than those with a widespread disease (7) and may benefit from salvage targeted therapies in the metastatic setting (8, 12).

In patients with PCa in whom distant metastases are suspected, whole-body imaging (WBI) (head, neck, torso, and the proximal part of the limbs) can be used to guide the treatments (8, 12). Among the available modalities, ^18^F-fluoride (NaF) positron emission tomography/computed tomography (PET/CT), ^18^F-fluorocholine (FCH) PET/CT, and whole-body magnetic resonance imaging (WBMRI) have been proposed for PCa metastasis detection (9, 10, 38). WBMRI enables the detection of lymph node metastases and distant metastases in one test (11). Multi-parametric MRI has a better performance than a classical bone scan and targeted X-ray for detecting bone metastasis (12) and might have better performance than PET/CT (13, 14). Choline PET/CT may have a better detection rate of bone metastases compared to bone scans at the initial staging or restaging after a biochemical recurrence in men with PCa (15). ^18^F-choline PET may have poor sensitivity but high specificity for bone metastasis detection in men with PCa (16).

A recent meta-analysis has compared the diagnostic performance in staging between WBMRI and PET/CT in patients with non-small cell lung cancer and showed no difference between the two imaging modalities (17). There are no guidelines specific to the diagnosis of bone metastases in PCa, and there are no meta-analyses comparing WBMRI and PET/CT in PCa.

Therefore, the aim of this meta-analysis was to compare WBMRI and PET/CT for bone metastasis detection in patients with PCa. The results could provide some guidance for the treatment strategy of patients with PCa.

## Materials and Methods

### Literature Search

This meta-analysis was conducted according to the Preferred Reporting Items for Systematic Reviews and Meta-Analyses (PRISMA) guidelines (18). Papers published up to April 2020 were searched for in PubMed, Embase, and the Cochrane library using the MeSH term “Prostatic Neoplasms,” and relevant keywords such as “whole-body magnetic resonance imaging.” The relevant articles were searched for using the PICO principle (19), followed by screening based on the eligibility criteria: (1) population: patients with untreated primary PCa who underwent WBMRI or PET/CT for bone metastasis detection; (2) interventions: both WBMRI and PET/CT for the diagnosis of bone metastases; (3) outcomes: the numbers of patients with true positive, false positive, false negative, and true negative results for WBMRI and PET/CT; (4) study type: focused on humans; and (5) language: limited to English.

### Data Extraction

The study characteristics (authors, year of publication, the country where the study was performed, type of study design, PSA levels, type of PET/CT, and sample size), treatment parameters (number of case analyses) were based on patients or lesions, standard reference per the study, and age of the patients, and primary outcomes (true positive, false positive, false negative, and true negative results for WBMRI and PET/CT) were extracted by two authors (Yuefu Zhan and Guangming Zhang) independently. Discrepancies were solved by the discussion.

### Quality of the Evidence

Four prospective cohort studies and one retrospective cohort study could be included. The quality assessment was conducted independently by two authors (Yuefu Zhan and Guangming Zhang) using the Quality Assessment of Diagnostic Accuracy Studies-2 (QUADAS-2) for this particular review (20). The risk of bias was evaluated using the risk of bias in non-randomized studies of interventions tool (ROBINS-I) (21). Discrepancies in the quality assessment were solved by discussion.

### Statistical Analysis

The summarized sensitivity, specificity, positive likelihood ratios (PLR), negative likelihood ratios (NLR), and diagnostic odds ratios (DOR) are presented with their corresponding 95% confidence intervals (CIs) and were obtained by means of a bivariate regression model using random effects based on the true positive and negative and false positive and negative rates in each study. The summary receiver operating characteristic (ROC) curve and the area under the curve (AUC) for WBMRI and PET/CT were calculated using a hierarchical regression model. The effect estimates and the corresponding 95% CIs of the diagnostic parameters were available for each study. The summary ratios between WBMRI and PET/CT and 95% CIs for sensitivity, specificity, PLR, NLR, DOR, and AUC were computed by random-effects models. The heterogeneity across the included studies was calculated using the *I*^2^ and Q statistic, and a *P* < 0.05 was regarded as significant heterogeneity. Two-sided *P* < 0.05 are considered statistically significant across the studies included. The statistical analyses were conducted using the MetaDiSc software (version 1.4) and STATA SE 14.0 software (StataCorp, College Station, TX, USA). No publication analysis could be performed because the number of included studies was <10 (22).

## Results

### Selection and Characteristics of the Studies

**Figure 1:**
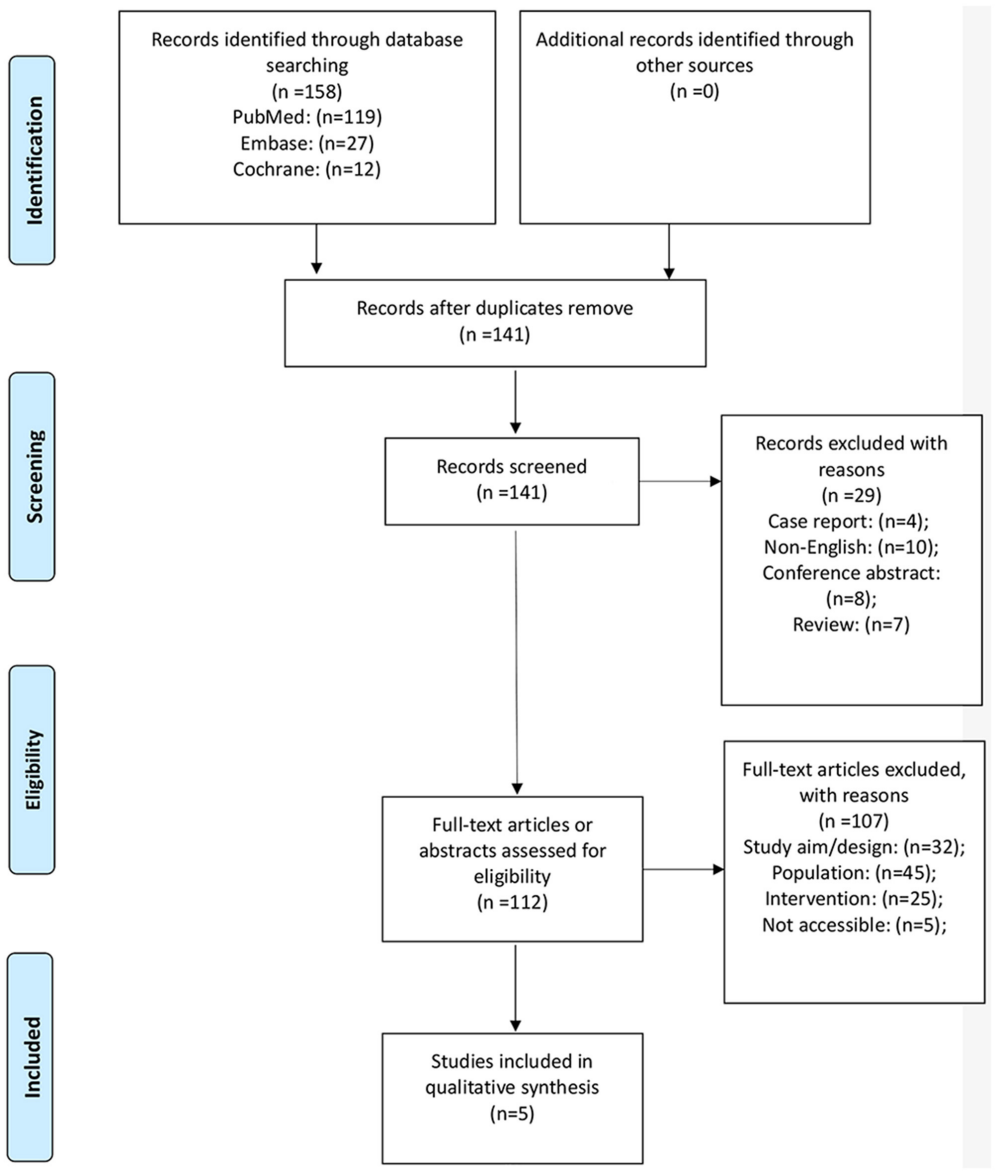
Study selection process.

Figure 1 presents the study selection process. A total of 158 records were initially identified, and 141 were examined after the duplicates were removed. Twenty-nine were preliminarily excluded, and 112 full-text articles were evaluated for eligibility. Among them, 107 were excluded (32 because of study aim/design, 45 because of the population, 25 because of the intervention, and five because they were not accessible). Therefore, five studies were included (23–27).

There are four prospective studies (24–27) and one retrospective study (23). Three studies are based on the patient (24–27) and two on the lesions (23, 26). The five studies included 657 patients. Table 1 presents the characteristics of the studies and the diagnostic values of WBMRI and PET/CT for each individual study. Two studies used ^11^C-choline-PET/CT, two used F-NaF-PET/CT, and one used Ga-PSMA-PET/CT.

**Table 1:** Characteristics of the studies included.

**References**	**Country**	**Study design**	**Base of analysis**	**Standard reference**	**Type of PET/CT**	**PSA, Median (range), ng/ml**	**Age, y**	**WBMRI**				**Control (PET/CT)**			
								**TP**	**TN**	**FP**	**FN**	**TP**	**TN**	**FP**	**FN**
Dyrberg et al. (27)	Denmark	PD	Patient	Determined as a panel diagnosis by three imaging specialists	^11^C-Choline-PET/CT	30 (51,000)	75 ± 9	16	29	6	4	20	35	0	0
Jambor et al. (25)	Finland	PD	Patient	Consensus based on all imaging modalities, follow-up data, and laboratory results	F-NaF PET/CT	/	/	8	18	1	0	7	19	1	0
Mosavi et al. (24)	Sweden	PD	Patient	Consensus based on all imaging modalities, follow-up data	F-NaF PET/CT	14 (1.3–950)	67 (57–80)	4	10	2	0	4	7	5	0
Wieder et al. (26)	Germany	PD	Lesion	Clinical follow-up and histopathology	^11^C-Choline-PET/CT	29.9 (1.0–670)	68 (54–80)	55	356	51	15	65	380	6	5
Eschmann et al. (23)	Germany	RD	Lesion	Consensus based on all imaging modalities, follow-up data, and laboratory results	Ga-PSMA-PET/CT	5.4 (0.15–200)	64.1 (51–79)	39	59	0	5	41	59	0	3

*WBMIR, whole-body magnetic resonance imaging; PTC/CT, positron emission tomography/computed tomography; TP, true positive; TN, true negative; FP, false positive, FN, false negative; PD, prospective study; RD, retrospective study*.

Table 2 presents the quality assessment of the studies included. One retrospective study (23) and three prospective studies (24–26) do not meet three criteria: avoidance of a case-control design, avoidance of inappropriate exclusions, and the use of a prespecified threshold. The study by Dyrberg et al. (27) meets only three criteria. For all five studies, it is uncertain whether the reference standard results were interpreted without knowledge of the results of the index test. Supplementary Table 1 presents the ROBINS-I evaluation.

**Table 2:** Quality evaluation of the included studies using the QUADAS-2 tool.

		**References**				
		**Eschmann et al. (23)**	**Mosavi et al. (24)**	**Jambor et al. (25)**	**Wieder et al. (26)**	**Dyrberg et al. (27)**
Patient selection	Was a consecutive or random sample of patients enrolled?	Y	Y	Y	Y	Y
	Was a case-control design avoided?	N	N	N	N	Y
	Did the study avoid inappropriate exclusions?	N	N	N	N	N
Index test(s)	Were the index test results interpreted without knowledge of the results of the reference standard?	Y	Y	Y	Y	U
	If a threshold was used, was it prespecified?	N	N	N	N	N
Reference standard	Is the reference standard likely to correctly classify the target condition?	Y	Y	Y	Y	N
	Were the reference standard results interpreted without knowledge of the results of the index test?	U	U	U	U	U
Flow and timing	Was there an appropriate interval between index test(s) and reference standard?	Y	Y	Y	Y	U
	Did all patients receive a reference standard?	Y	Y	Y	Y	U
	Did patients receive the same reference standard?	Y	Y	Y	Y	U
	Were all patients included in the analysis?	Y	Y	Y	Y	Y

### Sensitivity

**Figure 2:**
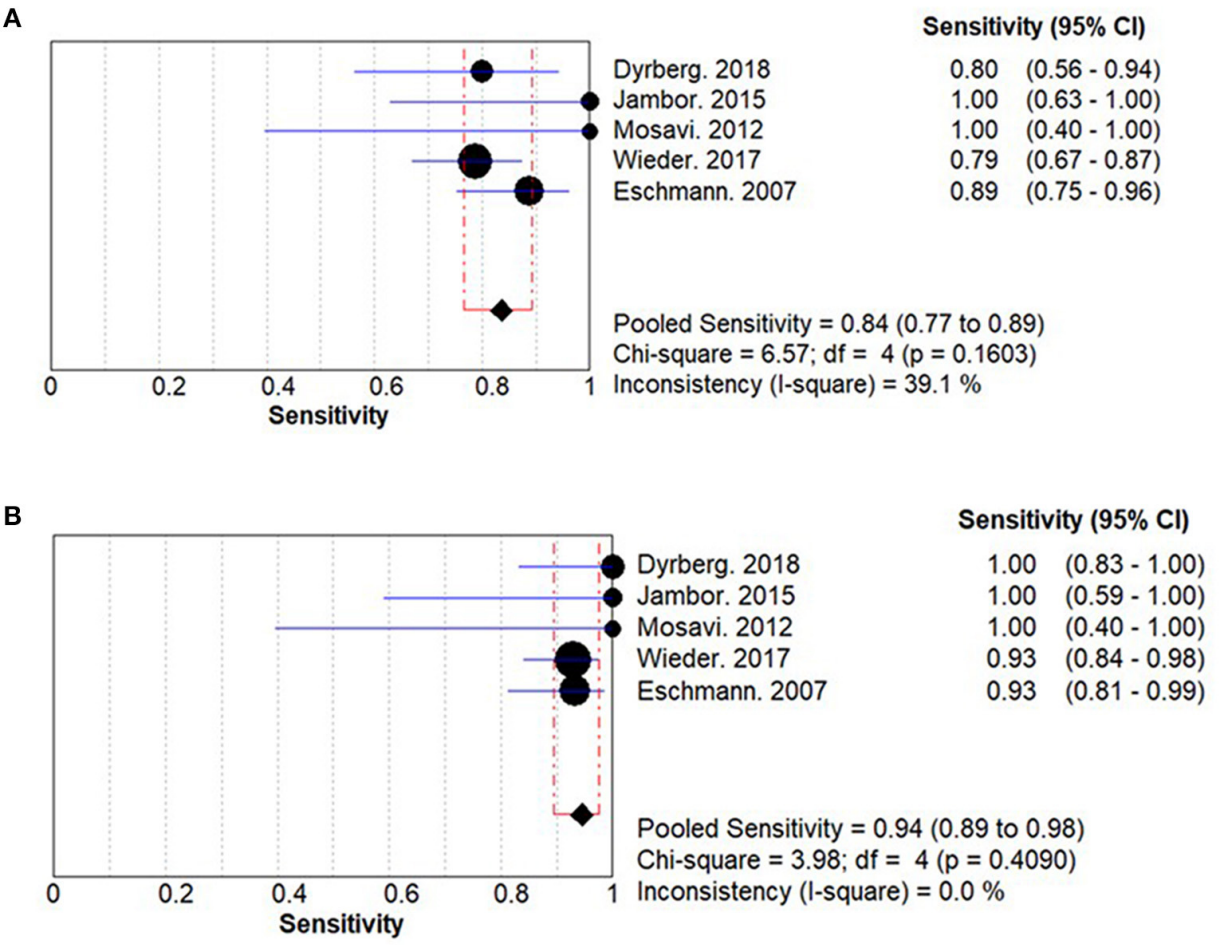
Summary results for sensitivity for whole-body magnetic resonance imaging (WBMRI) **(A)** and positron emission tomography/computed tomography (PET/CT) **(B)**.

The summary sensitivities for WBMRI and PET/CT for bone metastasis detection in PCa are 0.84 (95% CI: 0.77–0.89) and 0.94 (95% CI: 0.89–0.98), respectively (Figure 2). A significant difference is observed between WBMRI and PET/CT for sensitivity (ratio between WBMRI and PET/CT: 0.896; 95% CI: 0.813–0.987; *P* = 0.025; *I*^2^ = 0.0%, *P*_heterogeneity_ = 0.686) (Supplementary Figure 1; Table 3).

**Table 3:** Relative risk ratios between WBMRI and PET/CT for sensitivity, specificity, PLR, NLR, and DOR.

**Outcomes**	***N***	**Relative risk ratio (95% CI)**	***P***	***I*^**2**^ (%)**	***P* for heterogeneity**
Sensitivity	5	0.896 (0.813, 0.987)	0.025	0	0.686
Specificity	5	0.939 (0.855, 1.031)	0.184	78.8	0.001
PLR	5	0.416 (0.078, 1.031)	0.305	76.1	0.002
NLR	5	2.378 (1.127, 5.014)	0.023	0	0.476
DOR	5	0.130 (0.015, 1.108)	0.062	46.8	0.111

*PLR, positive likelihood ratio; NLR, negative likelihood ratio; DOR, diagnostic odds ratio*.

### Specificity

**Figure 3:**
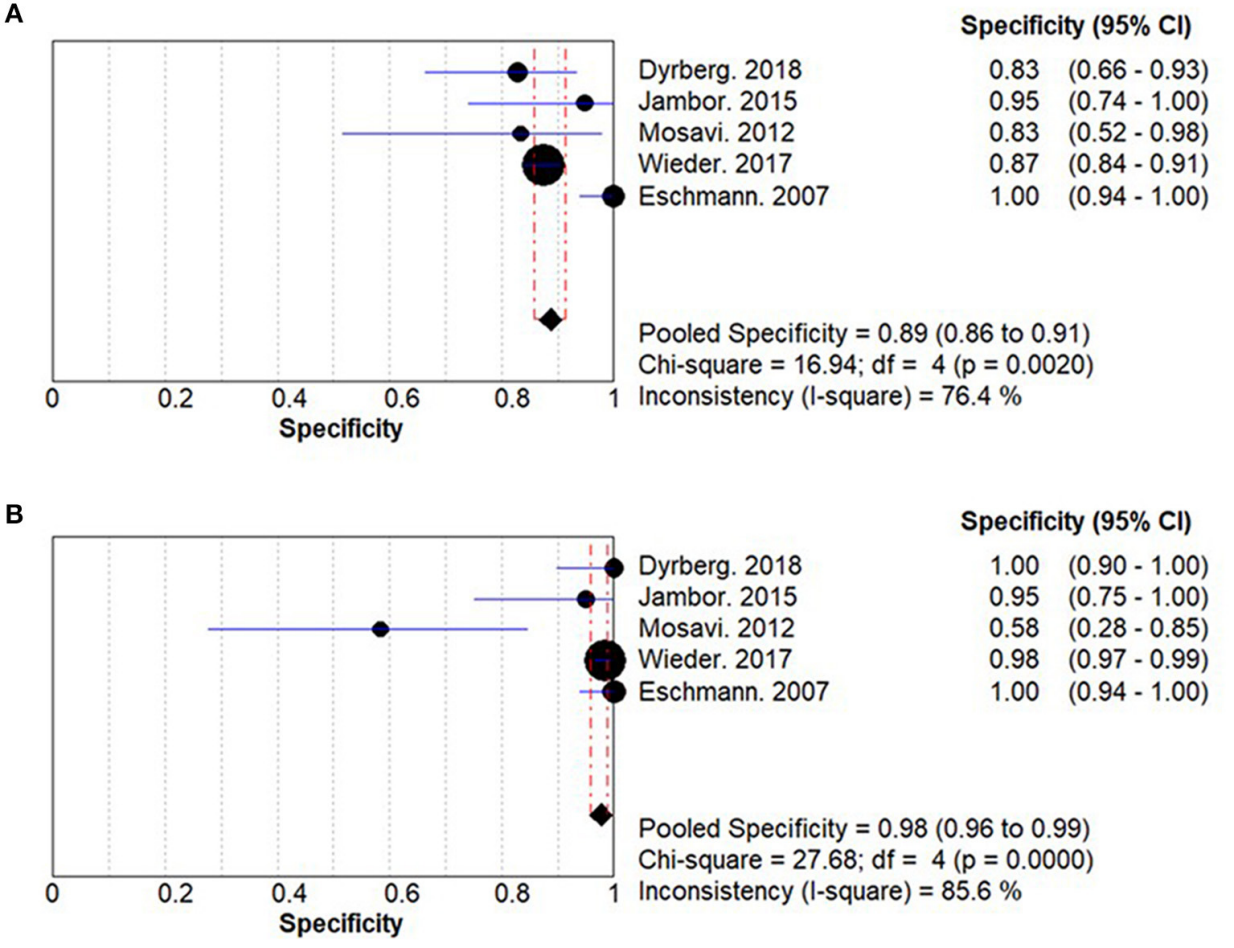
Summary results for specificity in whole-body magnetic resonance imaging (WBMRI) **(A)** and positron emission tomography/computed tomography (PET/CT) **(B)**.

The summary specificities for WBMRI and PET/CT for detecting bone metastases in PCa are 0.89 (95% CI: 0.86–0.91) and 0.98 (95% CI: 0.96–0.99), respectively (Figure 3). No significant difference is observed between WBMRI and PET/CT for specificity (ratio between WBMRI and PET/CT: 0.939; 95% CI: 0.855–1.031; *P* = 0.184; *I*^2^ = 78.8%, *P*_heterogeneity_ = 0.001) (Supplementary Figure 2; Table 3).

### Positive Likelihood Ratio

The summary PLRs for WBMRI and PET/CT for detecting bone metastases in PCa are 6.89 (95% CI: 3.59–13.25) and 23.39 (95% CI: 2.56–214.03), respectively (Supplementary Figure 3). WBMRI is not associated with a significant difference in PLR compared with PET/CT (ratio between WBMRI and PET/CT: 0.42; 95% CI: 0.08–2.22; *P* = 0.305; *I*^2^ = 76.1%, P_heterogeneity_ = 0.002) (Supplementary Figure 5; Table 3).

### Negative Likelihood Ratio

The summary NLRs for WBMRI and PET/CT for detecting bone metastases in PCa are 0.21 (95% CI: 0.14–0.29) and 0.07 (95% CI: 0.04–0.13), respectively (Supplementary Figure 4). WBMRI was associated with a significant difference in NLR compared with PET/CT (ratio between WBMRI and PET/CT: 2.38; 95% CI: 1.13–5.01; *P* = 0.023; *I*^2^ = 0.0%, P_heterogeneity_ = 0.476) (Supplementary Figure 6; Table 3).

### Diagnostic Odds Ratio

The summary DOR in WBMRI for detecting bone metastases of PCa is 44.93 (95% CI: 14.44–139.80; *I*^2^ = 47.2%, *P*_heterogeneity_ = 0.108) (Supplementary Figure 7). The DOR of PET/CT is 402.92 (95% CI: 70.93–2288.91; *I*^2^ = 51.3%, P_heterogeneity_ = 0.084) (Supplementary Figure 7). WBMRI has a similar a DOR compared with PET/CT (ratio between WBMRI and PET/CT: 0.13; 95% CI: 0.02–1.11; *P* = 0.062; *I*^2^ = 46.8%, P_heterogeneity_ = 0.111) (Supplementary Figure 8).

### ROC Analysis

The summary AUC for WBMRI is 0.88 (standard error: 0.032) and 0.98 (standard error: 0.013) for PET/CT for diagnosing bone metastases in PCa (Supplementary Figure 9).

### Discussion

A recent meta-analysis revealed no difference between WBMRI and PET/CT in non-small cell lung cancer (17), but no such study is available for PCa. Therefore, this meta-analysis aims to compare WBMRI and PET/CT for bone metastasis detection in patients with PCa. The results show that PET/CT presents a higher sensitivity and NLR for bone metastasis detection from PCa, whereas no differences are found for specificity and PLR, compared with WBMRI.

A previous meta-analysis of four studies that compared WBMRI and PET/CT for the detection of metastases from lung cancer showed that there are no differences in the diagnostic yield of WBMRI and PET/CT for the detection of the M status of lung cancer (17). A meta-analysis of MRI, choline-PET/CT, bone SPECT, and bone scintigraphy for the detection of bone metastasis from PCa showed that on a per-patient basis, MRI was better than choline-PET/CT and scintigraphy, while on a per-lesion basis, choline-PET/CT was better than bone SPECT and scintigraphy (14). That meta-analysis did not consider the N stage. Similar results were also suggested by a review by Pesapane et al. (28) in breast cancer. Importantly, that review suggested that WMBRI could be more sensitive than PET/CT for visceral metastases (28–30) and small hepatic and brain metastases (28, 31, 32), but WBMRI could be associated with more false-positives that PET/CT for bone metastases because bone marrow edema caused by benign lesions can appear as metastases on the apparent diffusion coefficient (ADC) map (28). A review highlighted that modern PET/CT protocols have a better diagnostic value than MRI for the detection of PCa metastases but that MRI still has a role to play (33). Since the present meta-analyses only examined bone metastases, this edema from benign lesions might explain, at least in part, why WBMRI fared less well than PET/CT. Nevertheless, other studies in patients with breast cancer reported a similar diagnostic value of WBMRI compared with ^19^F-FDG PET/CT for bone metastases (34), highlighting that the DWI maps must not be read alone but in combination with the morphological changes (28). Gutzeit et al. (35) reported better performance of WBMRI compared with PET/CT for skeletal metastases in PCa and breast cancer, while the SKELETA trial (25) reported equivalent diagnostic value for bone metastases from PCa. Those conflicting results can be due to the differences in imaging protocols, magnet strength, and radiologist experience among the different centers. Nevertheless, both WBMRI and PET/CT have been shown to be better than CT and bone scan in terms of sensitivity and specificity for bone metastases (36).

The results of this meta-analysis must be considered in light of its limitations. In one study (25), besides PCa, the authors also included patients with breast cancer for comparing the detection of bone metastases; for this meta-analysis, the data pertaining to PCa had to be extracted. Of the five included studies, the analyses are patient-based in three studies and lesion-based in two. The cancer stage for inclusion varied among studies. Among the five studies, three different PET/CT modalities were used. Several studies did not report the true/false positive/negative, and those numbers had to be estimated based on the reported information, such as sensitivity, specificity, PLR, NLR, and the total number of cases, using the Revman software. Regarding stratification based on the risk group, as the risk level of the included patients was not specifically defined in the included studies, and as the number of studies was small, any results in terms of the stratification of risk groups would probably not lead to firm conclusions. This study had heterogeneity, which could be due to different patient risk levels among the included studies and variations in guidelines and country-level practice.

In conclusion, PET/CT presents a higher sensitivity and NLR for the detection of bone metastases from PCa, whereas no differences are found regarding specificity and PLR compared with WBMRI. Although this meta-analysis suggests a possibly better diagnostic performance of PET/CT in the detection of bone metastases in patients with PCa compared with WBMRI, compared with PET/CT, WBMRI is less expensive, more available, less time-consuming, and radiation-free. Further high-quality studies comparing the diagnostic performance of various imaging modalities and optimizing the WBMRI protocols are still needed to improve metastasis early detection in patients with PCa in clinical practice. In addition, novel prostate-specific membrane antigen-based imaging modalities are being developed, further improving the detection of PCa metastases (37). Those modalities will have to be examined in the future.

## Data Availability Statement

The original contributions presented in the study are included in the article/Supplementary Material, further inquiries can be directed to the corresponding author/s.

## Author Contributions

YZ carried out the studies, participated in collecting data, and drafted the manuscript. YZ and ML performed the statistical analysis and participated in its design. GZ reviewed and helped to draft the manuscript. XZ provided data analysis and a lot of advice to interpretation to the results, which ensure the high quality of this paper. All authors read and approved the final manuscript.

## Conflict of Interest

The authors declare that the research was conducted in the absence of any commercial or financial relationships that could be construed as a potential conflict of interest.
