# Supplementary material for: Whole-Body MRI vs. PET/CT for the Detection of Bone Metastases in Patients With Prostate Cancer: A Systematic Review and Meta-Analysis
Source: Front Oncol. 2021 May 4;11:633833. doi: 10.3389/fonc.2021.633833 (PMC8130579; doi:10.3389/fonc.2021.633833)
Supplement: Supplementary file 1 [file Data_Sheet_1.docx]

Supplementary Material

# Supplementary Figures


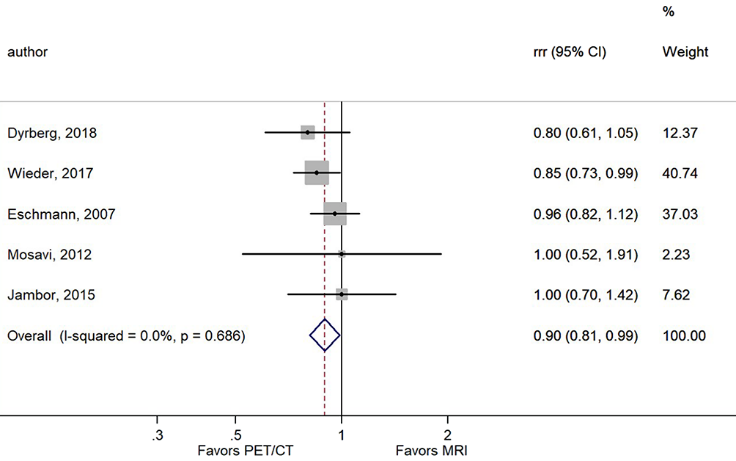


**Supplementary Figure S1.** The summary results for sensitivity between whole-body magnetic resonance imaging (WBMRI) and positron emission tomography/computed tomography (PET/CT).


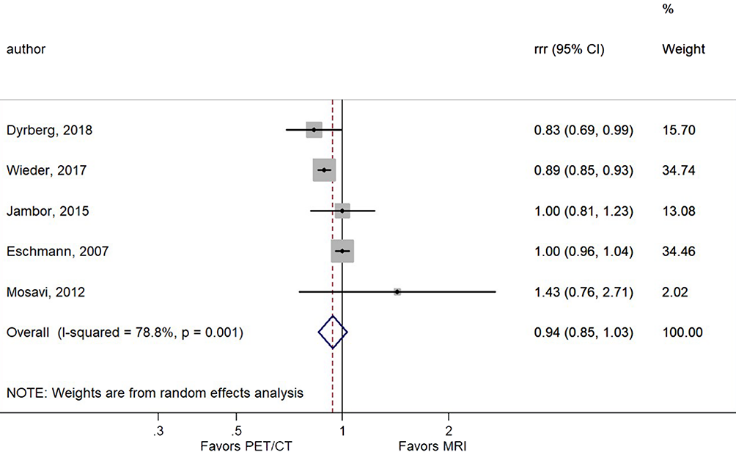


**Supplementary Figure S2.** The summary results for specificity between whole-body magnetic resonance imaging (WBMRI) and positron emission tomography/computed tomography (PET/CT).


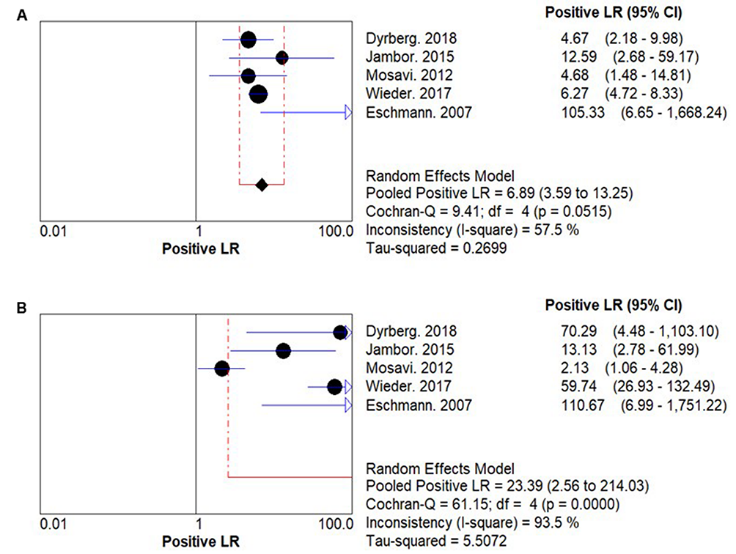


**Supplementary Figure S3.** The summary results for positive likelihood ratio (PLR) in whole-body magnetic resonance imaging (WBMRI) and positron emission tomography/computed tomography (PET/CT).


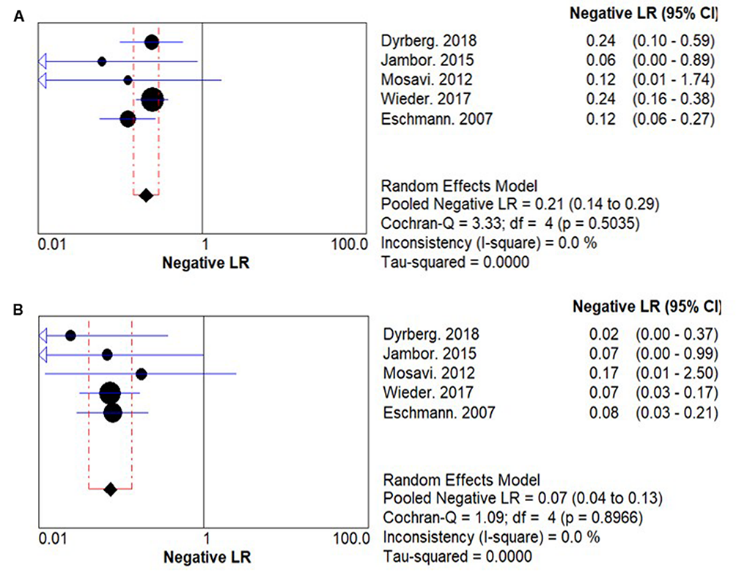


**Supplementary Figure S4.** The summary results for negative likelihood ratio (NLR) in whole-body magnetic resonance imaging (WBMRI) and positron emission tomography/computed tomography (PET/CT).


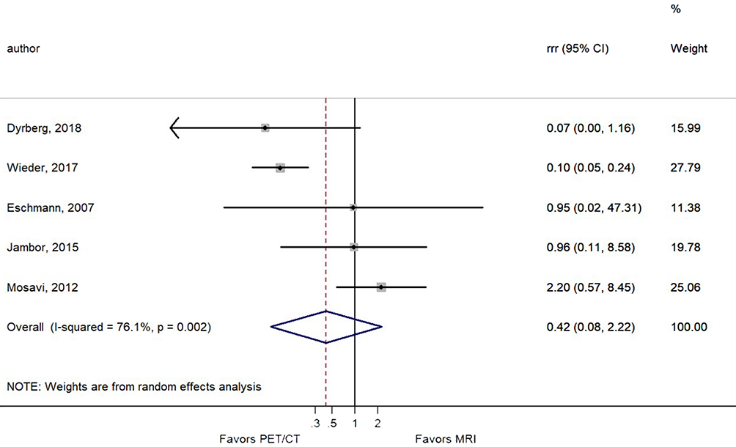


**Supplementary Figure S5.** The summary results for positive likelihood ratio (PLR) between whole-body magnetic resonance imaging (WBMRI) and positron emission tomography/computed tomography (PET/CT).


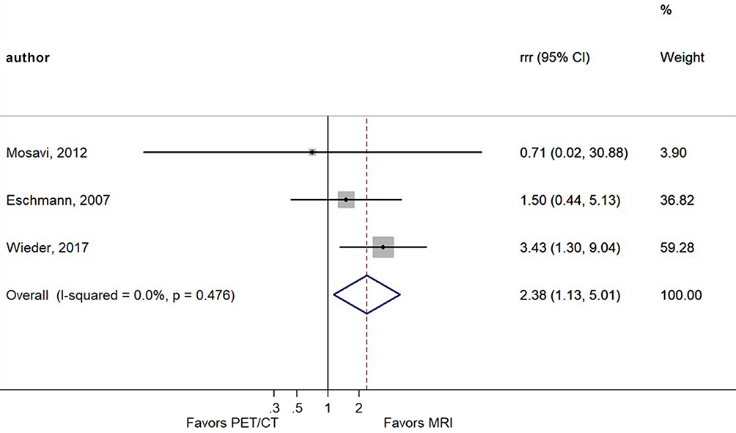


**Supplementary Figure S6.** The summary results for negative likelihood ratio (NLR) between whole-body magnetic resonance imaging (WBMRI) and positron emission tomography/computed tomography (PET/CT).


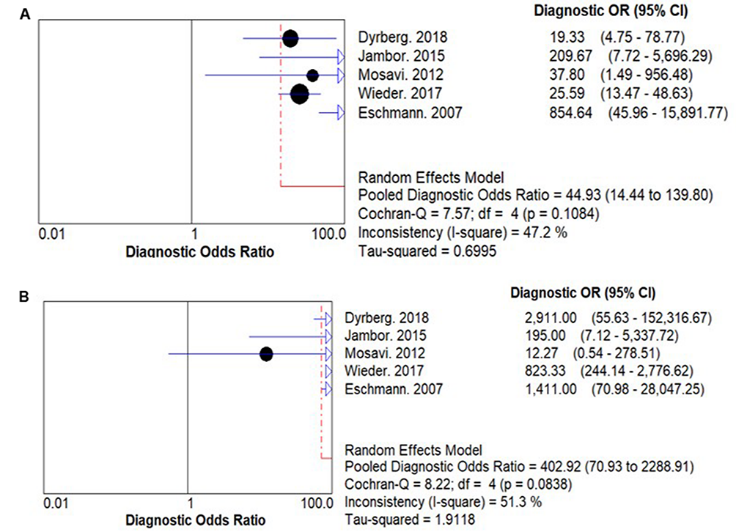


**Supplementary Figure S7.** The summary results for diagnostic odds ratio (DOR) in whole-body magnetic resonance imaging (WBMRI) and positron emission tomography/computed tomography (PET/CT).


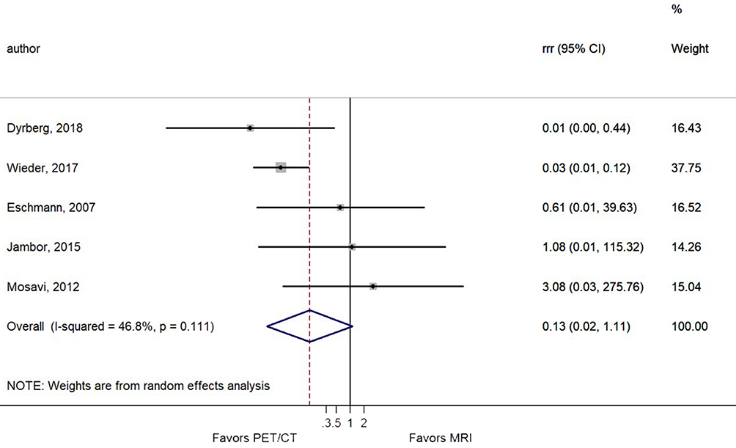


**Supplementary Figure S8.** The summary results for diagnostic odds ratio (DOR) between whole-body magnetic resonance imaging (WBMRI) and positron emission tomography/computed tomography (PET/CT).


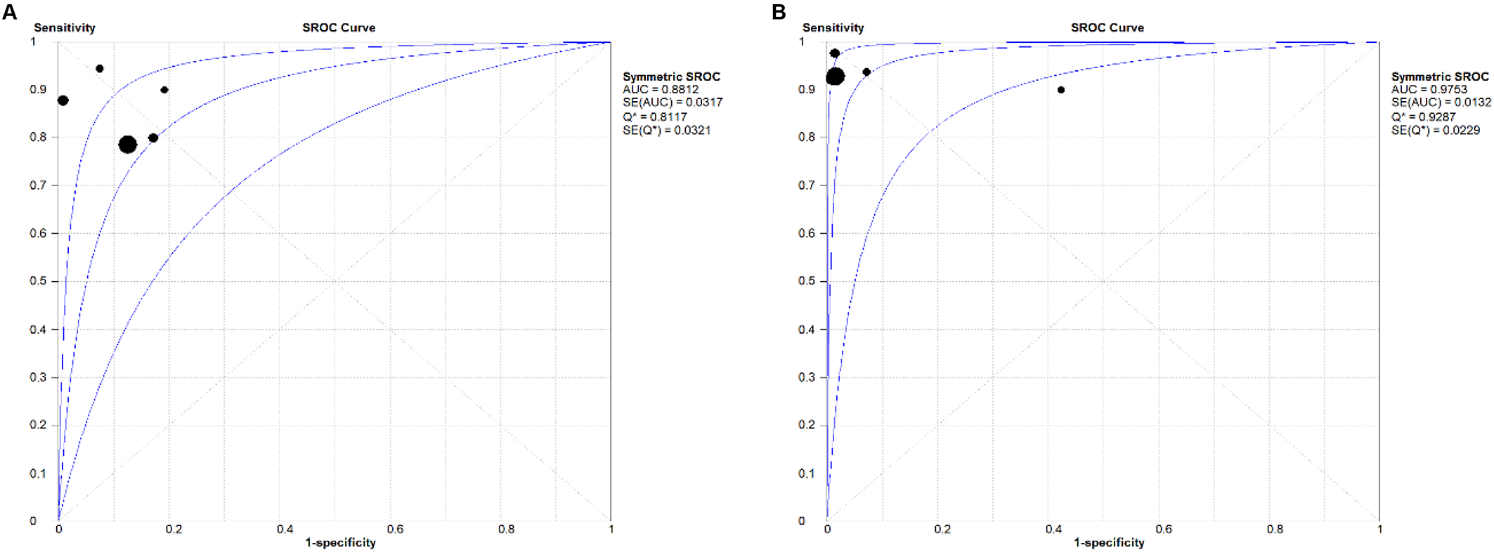


**Supplementary Figure S9.** The summary results for the area under the receiver operating characteristic (ROC) curves for whole-body magnetic resonance imaging (WBMRI) and positron emission tomography/computed tomography (PET/CT).

# Supplementary Table

**Supplementary Table S1.** Evaluation of the risk of bias using the Risk of bias in non-randomized studies of interventions tool (ROBINS-I)

| Study | Bias due to confounding | Bias in selection of participants into the study | Bias in classification of interventions | Bias due to deviations from intended interventions | Bias due to missing data | Bias in measurement of outcomes | Bias in selection of the reported result |
| --- | --- | --- | --- | --- | --- | --- | --- |
| Dyrberg, 2018 | Low | Low | Low | Low | Low | Moderate | Low |
| Jambor, 2015 | Low | Moderate | Low | Low | Low | Low | Low |
| Mosavi, 2012 | Low | Low | Low | Low | Low | Low | Low |
| Wieder, 2017 | Low | High | Low | Low | Low | Low | Low |
| Eschmann, 2007 | Low | High | Low | Low | Low | Low | Low |
